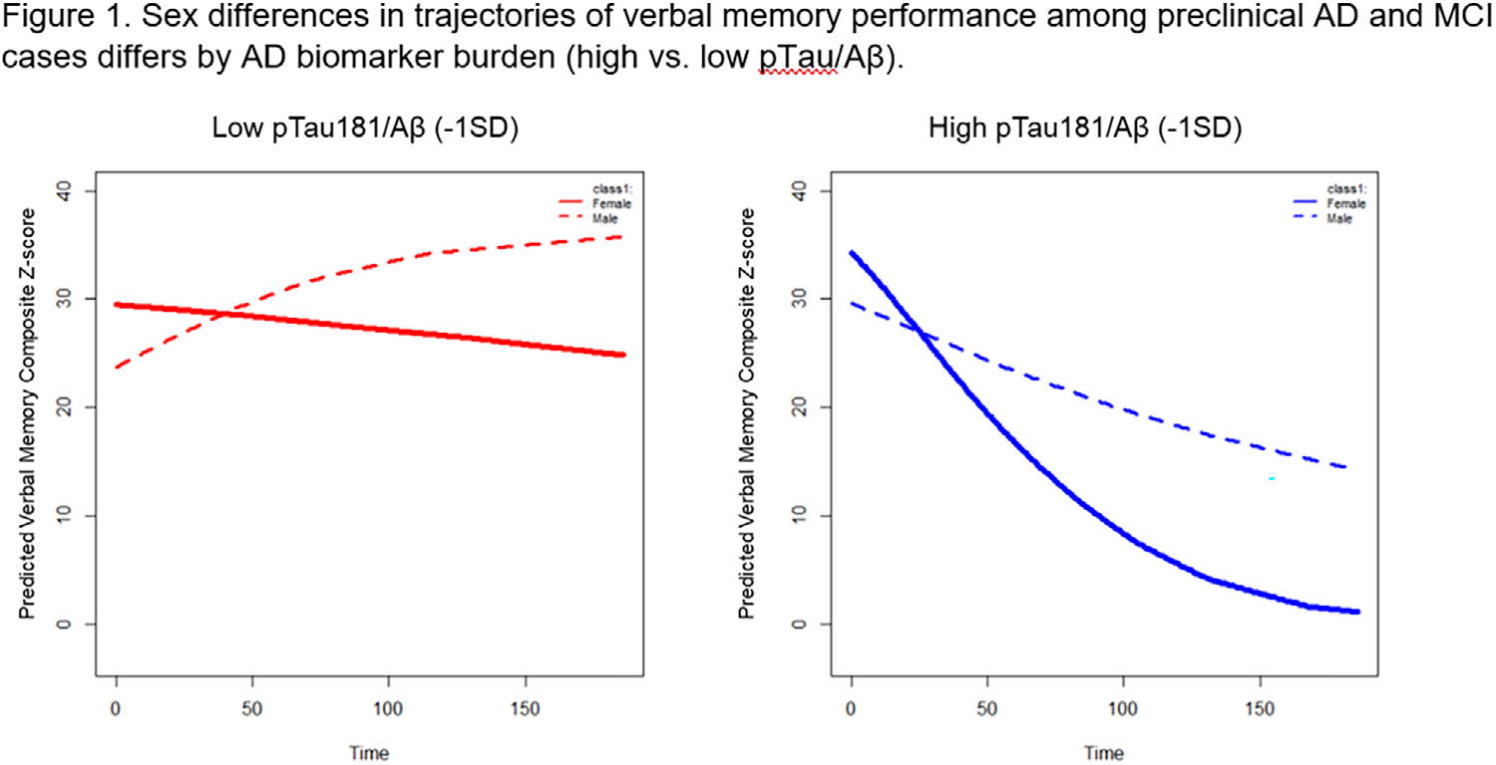# Sex Differences in the Clinical Manifestation of AD Pathology in Early‐Stage Alzheimer’s Disease

**DOI:** 10.1002/alz.095699

**Published:** 2025-01-09

**Authors:** Erin E. Sundermann, Sarah Banks, Mark W. Bondi, Maricedes Acosta Martinez, Anat Biegon, Lindsay J Rotblatt, Thomas Hildebrandt

**Affiliations:** ^1^ University of California, San Diego, La Jolla, CA USA; ^2^ VA San Diego Healthcare System, San Diego, CA USA; ^3^ Stonybrook University, New York, NY USA; ^4^ Swedish Collegium for Advanced Study, Uppsala, Uppsala Sweden; ^5^ Stony Brook University School of Medicine, Stony Brook, NY USA; ^6^ Mt. Sinai School of Medicine, New York, NY USA

## Abstract

**Background:**

Despite women showing greater pathological tau burden than men in the preclinical and mild cognitive impairment (MCI) stages of Alzheimer’s disease (AD), they show a cognitive advantage, particularly in verbal memory, followed by a steeper decline. Thus, the question of sex differences in the clinical manifestation of AD pathology is important, but underexamined. Among preclinical AD and MCI participants, we examined sex differences in the relationship of changes in cerebrospinal fluid (CSF) pTau181/Aβ42 (pTau/Aβ) ratio to changes in verbal memory.

**Methods:**

Participants included 504 older individuals (age range: 55‐90, mean age = 73.5±6.7, 95% non‐Hispanic White; mean follow‐up = 3.9±2.7 years) classified as preclinical AD (89 women, 88 men) or MCI (118 women, 176 men) at baseline in the Alzheimer’s Disease Neuroimaging Initiative. Preclinical AD was defined as cognitively normal but positive for CSF or PET Aβ or pTau biomarkers. MCI was defined by the Jak/Bondi criteria. Rey Auditory Verbal Learning Test‐Immediate and Delayed Recall z‐scores were averaged to create a verbal memory composite score. We applied latent class mixture modeling to trajectories of the verbal memory composite score, allowing for clustering of the growth trajectories into latent classes. We then examined the effect of sex, time‐varying pTau/Aβ ratio, time and disease stage (preclinical versus MCI) and their interactions on verbal memory composite while adjusting for education, age, and APOE4 status.

**Results:**

At baseline, women showed significantly better verbal memory performance (p<.01) and higher CSF pTau levels (p = .003), but the pTau/Aβ ratio did not differ by sex (p = .33). A 1‐class, non‐linear model provided optimal fit. A significant time x sex x pTau/Aβ interaction (p = .016) revealed a sex‐dependent influence of the pTau181/Aβ42 ratio on verbal memory over time, whereby, among those with higher pTau/Aβ ratio levels (>1SD above mean), higher pTau/Aβ ratio levels predicted verbal memory decline more strongly in women than in men.

**Conclusions:**

Findings suggest that**,** when AD pathology is more advanced, women show greater verbal memory decline as AD pathology progresses. Our findings provide insight into why sex differences in the clinical trajectory of AD vary by disease stage and raise questions as to the biological mechanisms underlying these sex differences.